# A Multivariant Surrogate Virus Neutralization Test Demonstrates Distinct SARS-CoV-2-Specific Antibody Responses in People Living with HIV after a Fourth Monovalent mRNA Vaccination or an Omicron Breakthrough Infection

**DOI:** 10.3390/diagnostics14080822

**Published:** 2024-04-16

**Authors:** David Niklas Springer, Simon Daller, Michael Knappik, Katja Prüger, Sylvia Hartl, Robab Breyer-Kohansal, Elisabeth Puchhammer-Stöckl, Judith Helene Aberle, Lukas Weseslindtner, Marie Kathrin Breyer

**Affiliations:** 1 Center for Virology, Medical University of Vienna, 1090 Vienna, Austria; david.springer@meduniwien.ac.at (D.N.S.); katja.prueger@meduniwien.ac.at (K.P.); elisabeth.puchhammer@meduniwien.ac.at (E.P.-S.); judith.aberle@meduniwien.ac.at (J.H.A.); 2 Department of Respiratory and Pulmonary Diseases, Vienna Healthcare Group, Clinic Penzing, 1140 Vienna, Austria; simon.daller@gesundheitsverbund.at (S.D.); michael.knappik@gesundheitsverbund.at (M.K.); sylvia.hartl@lunghealth.lbg.ac.at (S.H.); robab.breyer-kohansal@lunghealth.lbg.ac.at (R.B.-K.); marie-kathrin.breyer@gesundheitsverbund.at (M.K.B.); 3 Ludwig Boltzmann Institute for Lung Health, 1140 Vienna, Austria; 4 Faculty of Medicine, Sigmund Freud University, 1020 Vienna, Austria

**Keywords:** SARS-CoV-2, Omicron, antibodies, vaccination, PLWH, surrogate, neutralization

## Abstract

While neutralizing antibodies (nAbs) induced by monovalent severe acute respiratory syndrome coronavirus 2 (SARS-CoV-2) vaccinations are primarily directed against the wildtype (WT), subsequent exposure to the Omicron variants may increase the breadth of the antibodies’ cross-neutralizing activity. Here, we analyzed the impact of an Omicron breakthrough infection (BTI) or a fourth monovalent mRNA vaccination on nAb profiles in people living with human immunodeficiency virus (PLWH). Using a multivariant surrogate virus neutralization test (sVNT), we quantified nAbs in 36 three-times vaccinated PLWH, of whom 9 acquired a serologically confirmed Omicron BTI, 8 received a fourth vaccine dose, and 19 were neither infected nor additionally vaccinated. While nAbs against WT and Delta increased after the BTI and a fourth vaccination, a significant increase against BA.1, BA.2, and BA.5 was only observed after the BTI. However, there was no significant difference in nAb concentrations between the samples obtained after the BTI and fourth vaccination. In contrast, nAb levels were significantly lower in PLWH, who were neither infected nor additionally vaccinated after three vaccinations. Thus, our study demonstrates the suitability of a multivariant sVNT to assess hybrid humoral immunity after Omicron BTIs in PLWH vaccinated against SARS-CoV-2.

## 1. Introduction

Measuring antibody responses against severe acute respiratory syndrome coronavirus type 2 (SARS-CoV-2) is essential to quantify individual differences in virus-specific humoral immunity after vaccinations or previous infections. This particularly applies to specific patient cohorts like people living with human immunodeficiency virus (HIV) [1,2]. Indeed, recent studies have demonstrated that higher levels of SARS-CoV-2-specific antibodies (e.g., with distinct neutralizing capabilities and of various immunoglobulin classes) correlate with a reduced risk for reinfections with SARS-CoV-2 Omicron variants [3,4,5]. However, no specific threshold has been identified as a general protection correlate.

Notably, in people living with HIV (PLWH), the humoral immunity to SARS-CoV-2 mRNA vaccinations in terms of neutralizing capabilities and total levels of spike-specific antibodies may be weaker. However, the immune response to the vaccinations must not necessarily be impaired when the CD4+ T-cell counts are not significantly decreased [6,7,8,9,10,11,12]. Accordingly, PLWH with lower CD4+ T-cells or a discordant immune response to antiretroviral therapy display weaker antibody responses after SARS-CoV-2 vaccinations, calling for an exact antibody assessment and additional vaccinations in these individuals [13,14,15].

Since most commercial immunoassays have not been adapted to the changed spike (S) protein of Omicron and still use the ancestral S as the target antigen, the emergence of the Omicron variant has challenged the widespread use of such antibody assays [16,17]. This limitation particularly applies to quantifying the SARS-CoV-2-specific antibodies in individuals with hybrid immunity who have been previously infected with different variants or have received vaccinations with Omicron-adapted, bivalent vaccines [3,4,5,18].

Thus, live-virus neutralization tests (NTs) using the wildtype (WT), older SARS-CoV-2 variants like the Delta variant, and multiple Omicron subvariants (e.g., BA.1, BA.2, and BA.5) have been used to characterize individual profiles and the composition of cross-neutralizing antibodies, particularly in individuals after bivalent SARS-CoV-2 vaccinations or BTIs with Omicron variants [18,19,20]. However, only a few studies have performed such comprehensive analyses in PLWH [2,21].

However, live-virus neutralization tests (NTs) are laborious and require laboratories of biosafety level 3. Therefore, multivariant surrogate neutralization tests (sVNTs) have been initially established and widely applied to quantify the antibody-mediated binding inhibition between ACE2 and the receptor-binding domain (RBD) of different variants as the target antigens [2,9,22,23,24].

In this study, we used such a multivariant sVNT to analyze the profiles of nAbs directed against different SARS-CoV-2 Omicron subvariants in three groups of PLWH who had all been vaccinated three times with monovalent WT mRNA vaccines. One group received a fourth dose of the monovalent WT mRNA vaccine, another group had a breakthrough infection with an Omicron variant, and the third group did not receive a fourth vaccination and was not infected with SARS-CoV-2.

## 2. Materials and Methods

### 2.1. Patients and Samples

The study included 36 PLWH (6 females, 30 males; median age: 55 years, range: 40–77) who received a series of three monovalent SARS-CoV-2 mRNA vaccinations between January 2021 and December 2021. The first dose of the vaccine was administered between January 2021 and June 2021, the second dose between April 2021 and July 2021, and the third dose between November and December 2021. In all 36 individuals, there was no evidence of a previous SARS-CoV-2 infection, as indicated by testing for antibodies against the SARS-CoV-2 nucleocapsid (see below). Thirty PLWH received three doses of BNT162b (Comirnaty, BioNTech/Pfizer, New York, NY, USA), two individuals received a series of three doses of mRNA-1273 (Spikevax, Moderna, Cambridge, MA, USA), two subjects received two doses of BNT162b and one dose of mRNA-1273, and two individuals received two doses of mRNA-1273 and one dose of BNT162b.

After the third vaccination, serum samples were obtained from all 36 PLWH after a median interval of 54 days (range: 20–102). Notably, after this first serum sampling, 8 PLWH received a fourth (booster) dose of monovalent BNT162b on the same day (right after) the serum sample was obtained (group A), while the remaining 28 were not vaccinated a fourth time. Then, a second serum sample was obtained from all 36 PLWH at a median of 366 days (228–424) after the third vaccination. In the eight individuals vaccinated four times, the median interval between the fourth (booster) vaccination and the second serum sampling was 103 days (27–239), while the interval between the third and the fourth vaccination was 218 days (161–370).

Out of the 28 PLWH vaccinated only three times, 9 individuals reported that after the first serum sampling, they subsequently suffered from a PCR-confirmed breakthrough infection (BTI) with SARS-CoV-2 that occurred between February 2021 and November 2021 and only caused mild symptoms like fever, coryza, chills, and fatigue (group B). During this period, the Omicron variant, mainly the subvariants BA.1, BA.2, and BA.5, caused over 99% of all SARS-CoV-2 infections in Austria. The median interval between the third vaccination and the infection was 235 days (49–369), and the interval between the infection and the second serum sampling was 126 days (15–328). Notably, the SARS-CoV-2 infection could be verified in all these 9 PLWH by documented seroconversion of nucleocapsid-specific antibodies between the first and second serum samples using the Elecsys^®^ Anti-SARS-CoV-2 N immunoassay (Roche Diagnostics, Mannheim, Germany).

The other 17 PLWH who had been vaccinated three times and the 8 individuals vaccinated four times had no SARS-CoV-2 infection between collecting the first and second serum samples (group C). The absence of any SARS-CoV-2 infection was verified by the absence of nucleocapsid-specific antibodies in both serum samples (Elecsys^®^ Anti-SARS-CoV-2 N assay).

The 36 PLWH included in this study were part of a larger HIV cohort (*n* = 88), monitored using two rounds of serum sampling after the administration of at least three doses of SARS-CoV-2 mRNA vaccines. The 36 individuals were specifically selected for this study based on the availability of samples and clinical information, the number of performed vaccinations (three versus four), and the presence or absence of a BTI with the Omicron variant by anti-nucleocapsid antibodies.

The 36 PLWH received antiretroviral treatment with Biktarvy (*n* = 29), Triumeq (*n* = 3), Abacavir/Lamivudine/Isentress (*n* = 1, Abacavir/Lamivudine/Intelence (*n* = 1), Genvoya (*n* = 1), and Dovato (*n* = 1). The median CD4 numbers per μL at the first and second serum samplings were 595 per μL (149–1269) and 585 per μL (130–1182). HIV RNA was undetectable (below 30 copies/mL) in all the samples from all the individuals except for one subject, who displayed 46 copies/mL in the second serum sample.

Furthermore, the study included 15 non-HIV-infected individuals as controls who received four doses of SARS-CoV-2 mRNA vaccines (four doses of BNT162b: *n* = 10; three doses of BNT162b plus one dose of mRNA-1273: *n* = 5) and did not show any evidence of SARS-CoV-2 infection (nucleocapsid-specific antibodies undetectable). The median interval between the fourth (booster) vaccination and the serum sampling was 24 days (20–30).

### 2.2. Multivariant Surrogate Virus Neutralization Test

The multivariant surrogate virus neutralization assay was performed as described previously [25]. In brief, the sVNT is based on the commercial SARS-CoV-2 VoC ViraChip^®^ IgG microarray by Viramed (Planegg, Germany). However, to be used as an sVNT, the manufacturer plotted the RBD proteins of the SARS-CoV-2 wildtype (WT), the Delta variant, and the Omicron subvariants BA.1, BA.2, and BA.5 in triplets as the target antigens in each well. After incubation with the diluted serum samples, recombinant ACE2 bound to alkaline phosphatase (ACE2-AP, provided by Viramed) was added to each well. This ACE2 could only bind to the RBD proteins in inverse correlation to the levels of neutralizing antibodies against the specific SARS-CoV-2 variants and Omicron subvariants in the samples. After washing, the bound ACE2-AP (if present) was made visible by a colorimetric reaction of a chromogen substrate and assessed using the Viramed plate reader.

For each dilution step performed with each sample, the variant-specific ACE2-RBD-binding inhibition was calculated as a percentage of the reduction of the inhibited color reaction. Each sample was tested in five serial two-fold dilutions, starting from 1:20 up to 1:320. In some cases, where the measurements were still out of range at the dilution of 1:320 (i.e., inhibition of 100% of one or more antigens), additional dilutions were performed up to 1:2560.

Variant-specific ACE2-RBD-binding inhibition was first calculated as a percentage of the reduction of the inhibited color reaction relative to the maximum uninhibited color reaction in a negative buffer control sample (incubation without serum but with ACE2-AP) after subtraction of the background (i.e., 100% represents complete inhibition of the binding). The quantitative results were given in arbitrary units, which showed the strongest correlation with the live-virus NTs.

### 2.3. Statistical Analyses

The antibody levels (i.e., the levels of binding inhibition between ACE2 and RBD proteins from different SARS-CoV-2 variants) within individuals at different time points after the third SARS-CoV-2 mRNA vaccination, including before and after the fourth vaccination or after Omicron BTI, were compared using the Wilcoxon matched pairs test. The levels of ACE2-RBD-binding inhibition among the three-times vaccinated individuals, of whom the first group acquired Omicron BTI (group A), the second group was vaccinated a fourth time (group B), and the third group (group C) was neither vaccinated a fourth time nor acquired an Omicron BTI, were compared using the non-parametric Kruskal–Wallis test, followed by Dunn´s multiple comparison test. The intervals between the third vaccination and Omicron BTI (group A) and between the third and fourth vaccinations (group B) were compared using the Mann–Whitney *U* test. Furthermore, the Mann–Whitney *U* test was used to compare the levels of ACE2-RBD-binding inhibition between PLWH and non-HIV-infected individuals after four vaccinations. Alpha was set to 0.05, respectively. Statistical analyses were performed, and figures were created using GraphPadPrism version 9.5.1.

## 3. Results

### 3.1. Antibody Levels in PWLH after Three Doses of mRNA Vaccines

This study analyzed the breadth of neutralizing antibody responses in PLWH following an Omicron BTI (group A) or a fourth monovalent SARS-CoV-2 WT mRNA vaccination (group B). It also included PLWH vaccinated only three times without any serological evidence of a SARS-CoV-2 infection (group C).

**Figure 1:**
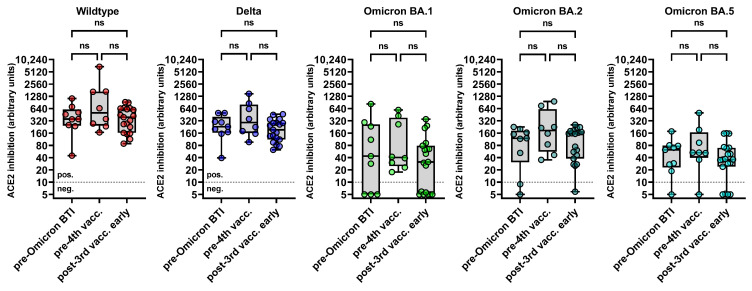
Baseline levels of binding inhibition between ACE and RBDs of different SARS-CoV-2 variants as assessed by the multivariant sVNT. The y-axis displays the levels of binding inhibition between angiotensin-converting enzyme 2 (ACE2) and the receptor-binding domains (RBD) of the SARS-CoV-2 wildtype, the Delta variant, and the Omicron subvariants BA.1, BA.2, and BA.5 in arbitrary units (calibrated to represent NT titers). These levels were compared among PLWH who were vaccinated three times with a monovalent mRNA vaccine, who subsequently acquired Omicron BTI, who received a fourth dose of the monovalent mRNA vaccine, and who were neither infected with SARS-CoV-2 nor vaccinated a fourth time. Comparisons were performed using the Wilcoxon matched pairs test (ns *p* ≥ 0.05). The cutoff for the sVNT was 10 arbitrary units for all variants, respectively. Abbreviations: BTI: breakthrough infection; ns: not significant.

First, the baseline levels of nAbs were quantified using a multivariant sVNT in all 36 three-times vaccinated PLWH. As shown in Figure 1, the levels of nAbs against SARS-CoV-2 WT, Delta, and Omicron BA.1, BA.2, and BA.5 were similar between the three groups.

### 3.2. Levels of ACE2-RBD-Binding Inhibition after Booster Vaccination versus Omicron Breakthrough Infection

Out of the 36 PLWH, 9 subjects who had not been additionally vaccinated acquired a SARS-CoV-2 infection between February 2021 and November 2021 (group A), while 8 individuals had received a fourth vaccination (group B). During this period, mainly the Omicron subvariants BA.1, BA.2, and BA.5 were circulating in Austria [26].

The intervals between the third vaccination and the BTI (group A, median of 235 days) and between the third and fourth vaccinations (group B, median of 218 days) did not significantly differ (*p* = 0.795; Supplementary Figure S1). Furthermore, there was no significant difference in the interval between the fourth vaccination and the BTI and the time point of the subsequent serum sampling (median of 126 days for group A versus a median of 106 days for group B, *p* = 0.943; Supplementary Figure S2).

**Figure 2:**
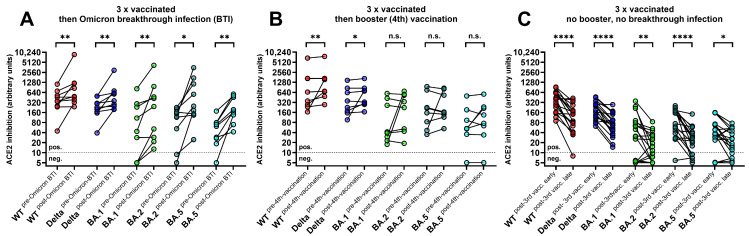
Levels of binding inhibition between ACE2 and RBDs of different SARS-CoV-2 variants as assessed by the multivariant sVNT. The y-axis displays levels of the binding inhibition between angiotensin-converting enzyme 2 (ACE2) and the receptor-binding domains (RBDs) of the SARS-CoV-2 wildtype, the Delta variant, and the Omicron subvariants BA.1, BA.2, and BA.5 5 in arbitrary units (calibrated to represent NT titers). These levels were compared within PLWH who were vaccinated three times with a monovalent mRNA vaccine, who (**A**) subsequently acquired Omicron BTI, (**B**) received a fourth dose of the monovalent mRNA vaccine, and (**C**) were neither infected with SARS-CoV-2 nor vaccinated a fourth time. Comparisons were performed using the Wilcoxon matched pairs test (**** *p* < 0.0001, ** *p* < 0.01, * *p* < 0.05, ns *p* ≥ 0.05). The cutoff for the sVNT was 10 arbitrary units for all the variants, respectively. Abbreviations: BTI: breakthrough infection; ns: not significant, WT: wildtype.

Figure 2A,B show the levels of ACE2-RBD-binding inhibition after Omicron BTI (group A) or a fourth mRNA vaccination (group B). In PLWH with Omicron BTI (Figure 2A), the levels increased against the WT, Delta (*p* < 0.0001 respectively), and Omicron BA.1 (*p* = 0.005), BA.2 (*p* < 0.0001), and BA.5 (*p* = 0.020). In contrast, a fourth monovalent mRNA vaccine dose (Figure 2B) increased the levels of ACE2-RBD-binding inhibition for the WT and the Delta variant (*p* = 0.008 and *p* = 0.039, respectively) but not for Omicron BA.1, BA.2, and BA.5. In three-times vaccinated PLWH who were neither vaccinated a fourth time nor infected with SARS-CoV (Figure 2C), the levels of ACE2-RBD-binding inhibition for the WT, Delta, and Omicron BA.1m, BA.2, and BA.5 significantly decreased over time (with *p* values <0.05 respectively). When the levels of ACE2-RBD-binding inhibition in four-times vaccinated PLWH were compared to the levels in four-times vaccinated individuals without HIV infection, no statistically significant difference was observed (Supplementary Figure S3).

### 3.3. Levels of ACE2-RBD-Binding Inhibition for WT, Delta, Omicron BA.1, BA.2 and BA.5

**Figure 3:**
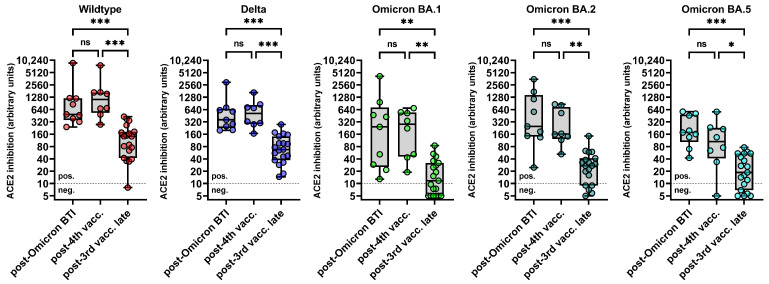
Levels of binding inhibition between ACE and RBDs of different SARS-CoV-2 variants post-booster vaccination, post-BTI, and late during follow-up. The y-axis displays the binding inhibition between angiotensin-converting enzyme 2 (ACE2) and the receptor-binding domains (RBDs) of the SARS-CoV-2 wildtype, the Delta variant, and the Omicron subvariants BA.1, BA.2 and BA.5 in arbitrary units (calibrated to represent NT titers). The median level (columns) and individual levels (dots) of ACE2-RBD-binding inhibition are shown as assessed by the sVNT in the three-times vaccinated PLWH (monovalent mRNA vaccines) from (1) group A after they acquired breakthrough infection (BTI) with an Omicron variant (“post-Omicron BTI”), (2) group B after they received a fourth dose of the monovalent WT vaccine (“post-booster”), and (3) group C who were neither vaccinated a fourth time nor infected late after the third vaccination (“post-3rd vacc. late”). Comparisons were performed using the Kruskal–Wallis test and Dunn´s multiple comparison tests. The cutoff for the sVNT was 10 units for all variants respectively. Abbreviations: BTI: breakthrough infection; (*** *p* < 0.001, ** *p* < 0.01, * *p* < 0.05, ns *p* ≥ 0.05). Abbreviations: BTI: breakthrough infection; vacc.: vaccination; ns: not significant.

Figure 3 shows the levels of ACE2-RBD-binding inhibition for the WT, Delta, and Omicron BA.1, BA.2, and BA.5 in all groups of the PLWH. Although the Omicron BTI induced a more substantial increase in these levels for BA.1, BA.2, and BA.5 than the fourth monovalent mRNA vaccination, there was no significant difference in the absolute levels for the Omicron subvariants among these groups. In addition, there was no difference in the levels of ACE-2-RBD-binding inhibition for the WT and the Delta variant among PLWH with four vaccinations and Omicron BTI (Figure 3).

As expected, the levels of ACE2-RBD-binding inhibition for all the SARS-CoV variants and Omicron subvariants were significantly lower in three-times vaccinated PLWH without Omicron BTI than those who received a fourth vaccine dose or acquired Omicron BTI (Figure 3).

## 4. Discussion

This study analyzed the levels of binding inhibition between ACE-2 and the RBDs of multiple SARS-CoV-2 variants and Omicron subvariants as a surrogate for cross-neutralizing antibodies. These levels were compared among three-times vaccinated PLWH (monovalent mRNA vaccines), of whom one part acquired an Omicron BTI, one part received a fourth dose of the vaccine, and one part was neither infected nor vaccinated a fourth time. The results of these analyses demonstrated a statistically significant increase in cross-neutralizing antibodies against BA.1, BA.2, and BA.5 only in PLWH after Omicron BTI but not after vaccination. However, there was no significant difference between the levels of nAbs after a fourth monovalent vaccination and Omicron BTI. In contrast, the nAb levels were significantly lower in three-times vaccinated PLWH who were neither vaccinated nor infected. This observed difference among three- and four-times vaccinated individuals thus highlights the beneficial effect of an additional SARS-CoV-2 booster dose on the total levels of nAbs regardless of their variant-specific neutralizing activity.

However, this study also showed that in PLWH, a fourth dose of a monovalent mRNA vaccine mainly increased nABs against the WT and Delta. At the same time, a BTI with the Omicron variant triggered a robust booster of nAbs against all variants, including Omicron BA.1, BA.2, and BA.5. As expected, a fourth vaccination and an Omicron BTI induced significantly higher levels of nAbs against all variants than in the three-times vaccinated PLWH who were neither vaccinated a fourth time nor infected. Furthermore, our observation that nAbs against the WT similarly increased after monovalent vaccination and BTI indicates that the establishment of hybrid humoral immunity in PLWH can only be assessed by multivariant sVNTs or variant-specific live-virus NTs and not by immunoassays containing only the WT as the target antigen. In a recent study, we similarly applied the sVNT in non-HIV-infected individuals and demonstrated higher nAb levels against BA.5 and BA.1 in subjects who had been vaccinated with the respective Omicron-adapted bivalent vaccines compared to individuals who had received monovalent wildtype vaccinations [25].

Indeed, multiple studies used anti-spike antibody assays and live-virus NTs containing the WT to quantify humoral immune responses to infections and monovalent vaccinations in PLWH and demonstrated that antibody levels in immunological responders to antiretroviral treatment were similar to non-HIV-infected individuals [6,7,8,9,10,11,12]. Conversely, low antibody concentrations were found in PLWH with low CD4 numbers and a discordant immune response to retroviral therapy [13,15]. A significant reduction in the neutralizing activity of antibodies induced by monovalent vaccines was demonstrated by studies using live-virus NTs comparing Omicron BA.1 to the WT. However, only a limited number of studies analyzed the composition of nAbs against different Omicron subvariants after BTIs or the application of Omicron-adapted bivalent vaccinations in PLWH [7,12,27].

In one study, Lapointe et al. measured nAbs against BA.1 and BA.5 in three-times vaccinated PLWH. The authors demonstrated that BTIs with the Omicron variants BA.1 or BA.2 triggered a robust booster of nAbs against the WT and BA.1. However, nAbs against BA.5 increased due to the BTIs, but this response was weaker than the ones against the WT and BA.1 [2]. The findings of our study correspond to these previous data on a variant-specific booster effect since we also observed a specific increase in cross-neutralizing activity after probable infection with Omicron BA.1, BA.2, and BA.5. Notably, Lapointe and colleagues demonstrated that the observed booster effect on hybrid immunity was comparable to non-HIV-infected controls [2].

It has recently been demonstrated by Vergori et al. that vaccination with an adapted WT/BA4/BA5 vaccine also increased the breadth of cross-neutralizing antibodies similar to an Omicron BTI [21]. In this study, the authors investigated PLWH who had CD4 levels < 200 cells/mL or previous AIDS using multiple variant-specific live-virus NTs and demonstrated that vaccination with an Omicron-adapted vaccine induced a similar increase of nAbs against the WT and BA.5 in previously SARS-CoV-2-infected and non-infected PLWH [21].

Our study certainly has the limitation of a relatively small study cohort compared to previous studies, so our observations should be regarded as preliminary. Furthermore, we used a sVNT that only included RBDs of Omicron BA.1, BA.2, and BA.5 and not younger variants like BQ.1.1 and XBB.1.

Nonetheless, our data support that after four SARS-CoV-2 mRNA vaccinations, nAb levels do not differ among PLWH and non-HIV-infected individuals when CD4+ T-cell counts in PLWH are not significantly decreased. Furthermore, our study demonstrates the suitability of multivariant sVNTs for assessing hybrid humoral immunity and nAB profiles against different SARS-CoV-2 variants in PLWH.

## Data Availability

The data from this study have not been publicly archived.

## Supplementary Materials

The following supporting information can be downloaded at https://www.mdpi.com/article/10.3390/diagnostics14080822/s1, Figure S1. Intervals between the 3rd vaccination and Omicron breakthrough infection (BTI; group A) and between 3rd and 4th vaccination (group B); Figure S2. Intervals between the Omicron breakthrough infection (BTI; group A) and the time point of serum sampling and between 4th vaccination (group B) and serum sampling; Figure S3. Levels of ACE2-RBD-binding inhibition as assessed by the sVNT in PLWH and non-HIV-infected individuals without SARS-CoV-2 infection after four monovalent SARS-CoV-2 mRNA vaccinations.
