# Supplementary material for: A Multivariant Surrogate Virus Neutralization Test Demonstrates Distinct SARS-CoV-2-Specific Antibody Responses in People Living with HIV after a Fourth Monovalent mRNA Vaccination or an Omicron Breakthrough Infection
Source: Diagnostics (Basel). 2024 Apr 16;14(8):822. doi: 10.3390/diagnostics14080822 (PMC11049121; doi:10.3390/diagnostics14080822)
Supplement: Supplementary file 1 [file diagnostics-14-00822-s001.zip › diagnostics-2908367-supplementary.pdf]

# Supplementary information

## Supplementary Figure S1

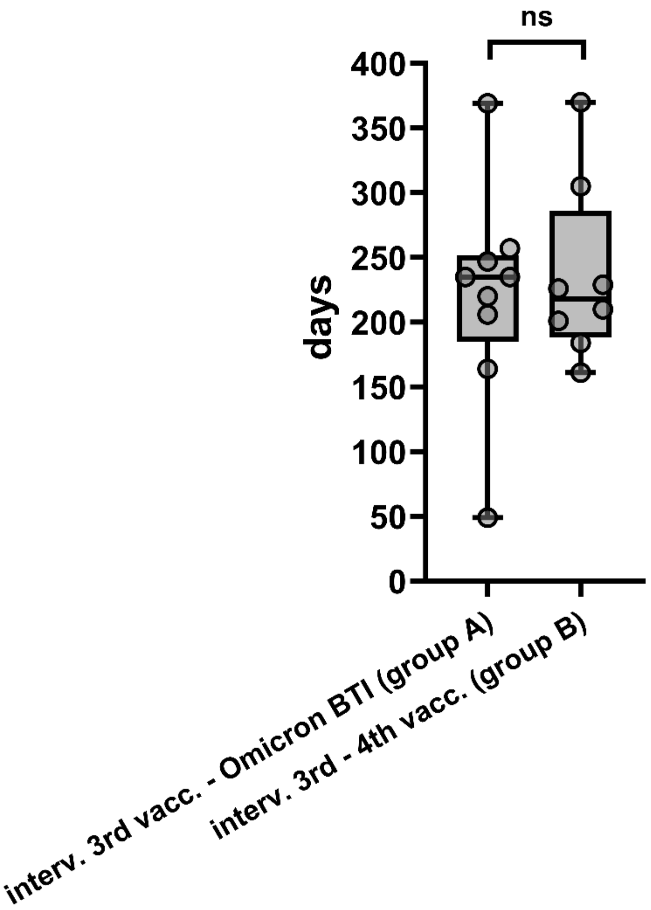


**Supplementary Figure S1. Intervals between the 3rd vaccination and Omicron breakthrough infection (BTI; group A) and between 3rd and 4th vaccination (group B)**

The y-axis displays the interval between the 3rd vaccination and Omicron breakthrough infection (BTI; group A; left) and between the 3rd and 4th vaccination (group B; right) in days. The median intervals are shown as columns, and the individual intervals are shown as dots. The intervals between the 3rd vaccination and Omicron BTI (group A) were compared with those between the 3rd and 4th vaccination (group B) using the Mann-Whitney U test. The difference was not statistically significant ($p=0.7954$); ns $p \geq 0.05$. Abbreviations: BTI: breakthrough infection; interv.; interval; vacc.: vaccination; ns: not significant.

**Supplementary Figure S2**

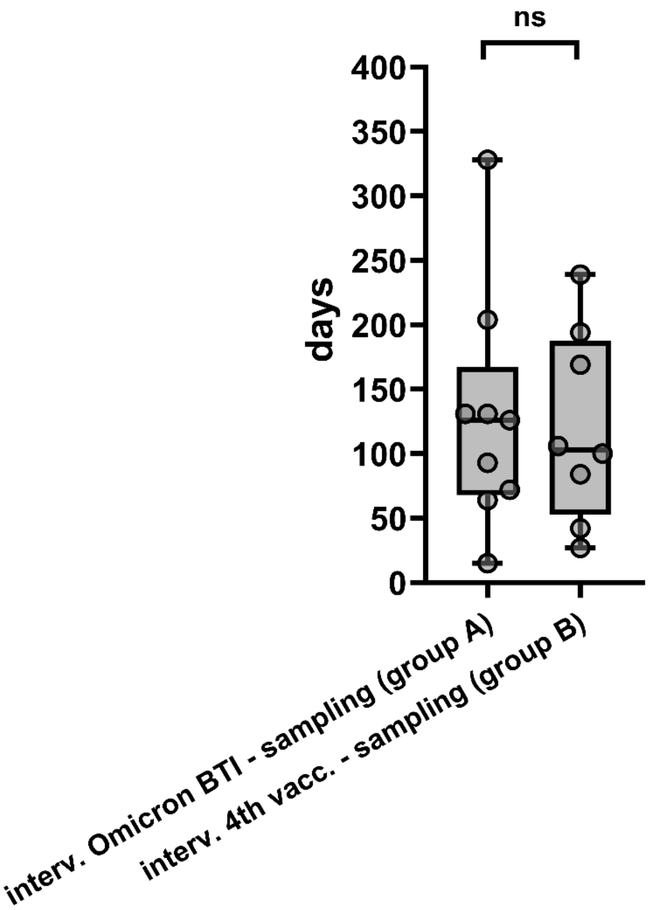


**Supplementary Figure S2. Intervals between the Omicron breakthrough infection (BTI; group A) and the time point of serum sampling and between 4th vaccination (group B) and serum sampling**

The y-axis displays the interval between the Omicron breakthrough infection (BTI; group A; left) and the time point of serum sampling and between the 4th vaccination and serum sampling (group B; right) in days. The median intervals are shown as columns, and the individual intervals are shown as dots. The intervals between Omicron BTI and serum sampling (group A) were compared with those between 4th vaccination and serum sampling (group B) using the Mann-Whitney U test. The difference was not statistically significant (p=0.9426); ns $p \geq 0.05$. Abbreviations: BTI: breakthrough infection; interv.; interval; vacc.: vaccination; ns: not significant.

**Supplementary Figure S3**

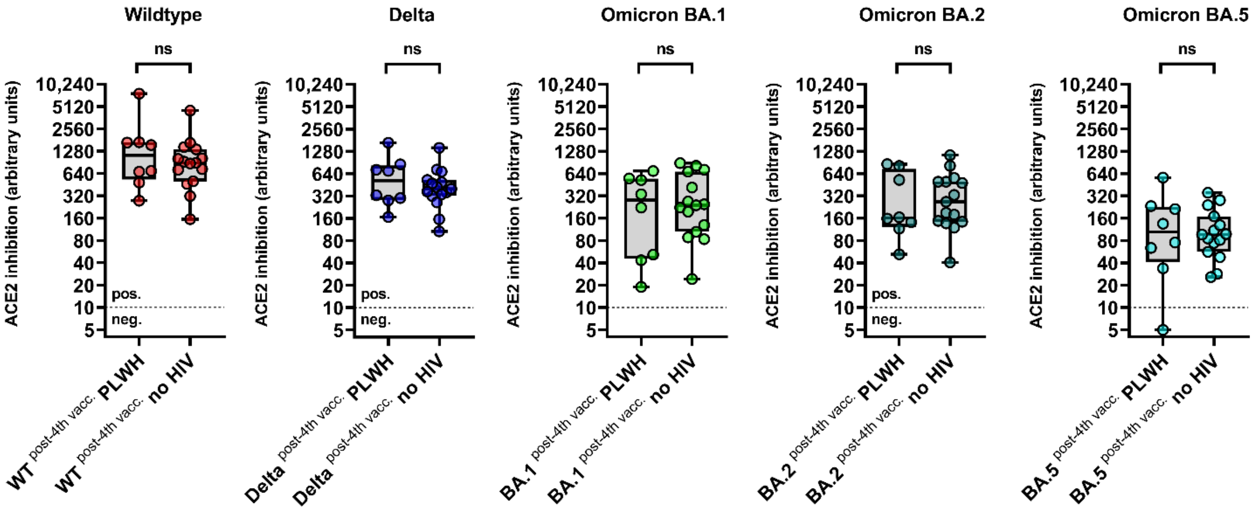


**Supplementary Figure S3. Levels of ACE2-RBD-binding inhibition as assessed by the sVNT in PLWH and non-HIV-infected individuals without SARS-CoV-2 infection after four monovalent SARS-CoV-2 mRNA vaccinations**

The y-axis displays the level of binding inhibition between angiotensin-converting enzyme 2 (ACE2) and the receptor-binding domains (RBDs) of the SARS-CoV-2 wildtype, the Delta variant, and the Omicron subvariants BA.1, BA.2, and BA.5 5 in arbitrary units (calibrated to represent NT titers). The Mann-Whitney-U test was used to compare levels of ACE2-binding inhibition among PLWH and non-HIV-infected individuals without any evidence of SARS-CoV-2 infection after four monovalent SARS-CoV-2 vaccinations. The differences were not statistically significant ($p \geq 0.05$, respectively). The cutoff for the sVNT was 10 arbitrary units for all variants respectively. Abbreviations: HIV: Human Immunodeficiency Virus, ns: not significant, PLWH: people living with HIV infection, ns: not significant, WT: wildtype.
